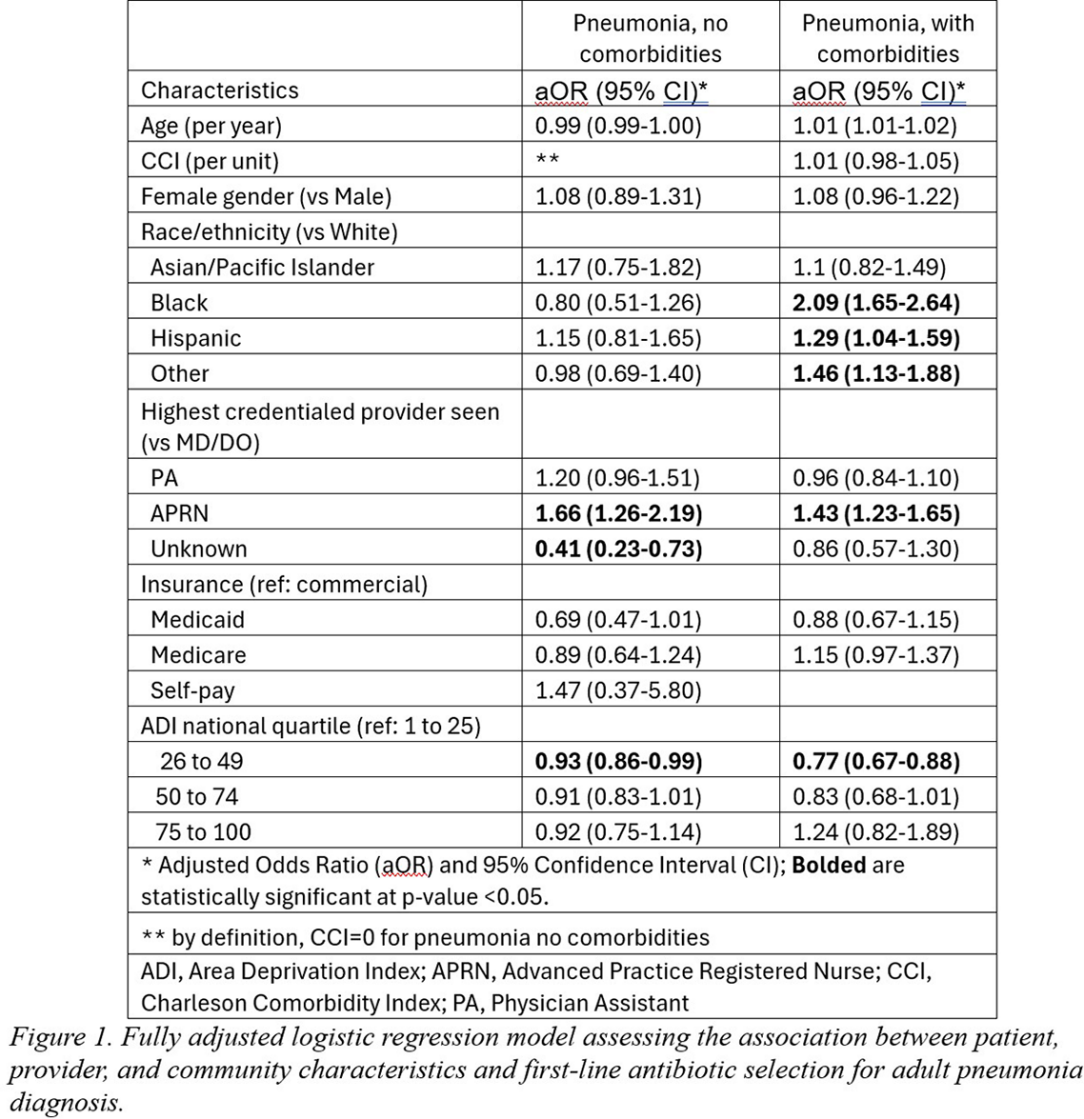# 90 Assessing the Viability of Indicator Organisms in Healthcare Ice Machines: Prevalence of Typical Hospital-Acquired Infections

**DOI:** 10.1017/ash.2026.10515

**Published:** 2026-06-23

**Authors:** Zoey Hall, Adovich Rivera, Susheel Reddy, Salva Balbale, Muhammad Dhanani, Katie Suda, Jasmine Marcelin, Mackenzie Keintz, Deja Glover, Geneva Wilson, Charlesnika Evans

**Affiliations:** 1 Northwestern University; 2 Division of Infectious Diseases, Feinberg School of Medicine, Northwestern University; 3 University of Pittsburgh School of Medicine; 4 University of Nebraska Medical Center; 5 Northwestern Medicine; 6 Edward Hines Jr. VA Hospital; 7 Northwestern University / Department of Veterans Affairs

## Abstract

**Background:** Guideline-concordant, first-line antibiotic prescribing is a core antimicrobial stewardship strategy to reduce unnecessary broad-spectrum use, antibiotic resistance, and adverse events. Community-acquired pneumonia (CAP) is a common indication for outpatient antibiotics, yet first-line prescribing patterns and equity remain understudied. This analysis assessed associations between first-line prescribing and patient, clinician, and community characteristics in urgent care settings. **Methods:** Electronic health records from 28 urgent care clinics within an integrated academic healthcare system (January 2023–April 2025) were analyzed. Adults (18+ years) with pneumonia diagnoses and at least one prescribed antibiotic were included. ‘Community-acquired pneumonia’ refers to pneumonia diagnosed in urgent care, though some cases may not meet formal CAP criteria. Episodes were classified by first-line use (CAP without comorbidities: amoxicillin or doxycycline; CAP with comorbidities: either amoxicillin/clavulanate plus doxycycline or a macrolide, or respiratory fluoroquinolone monotherapy). Cluster-adjusted logistic regression assessed differences in first-line prescribing by patient, clinician, and community factors. **Results:** Among 9,670 episodes (4,966 no comorbidities), first-line prescribing occurred in 90.8% of CAP with no comorbidities and 54.4% of CAP with comorbidities. Mean age was 48.4 years (SD 17.4) for patients without comorbidities and 60.7 years (SD 17.5) for patients with comorbidities; 23% and 48% of episodes occurred among adults ≥65 years. The most common non–first-line antibiotics were amoxicillin-clavulanate and clindamycin, for CAP with and without comorbidities respectively. For CAP without comorbidities, fully adjusted models showed higher odds of first-line prescribing for episodes managed by Advanced Practice Registered Nurses (APRNs) compared to physicians and lower odds among patients with higher neighborhood disadvantage (ADI 26–49 vs 1–25 percentile) as seen in Figure 1. The Area Deprivation Index (ADI) quantifies neighborhood socioeconomic disadvantage (1-100 is least to most disadvantaged). For CAP with comorbidities, Black and Hispanic patients (vs. White) and those treated by APRNs (vs. physicians) had higher odds of receiving first-line antibiotics, while patients in neighborhoods with higher ADI scores had lower odds (vs. 1–25 percentile; Figure 1). Across both groups, no differences were observed by age, gender, or payer. **Conclusion:** First-line prescribing for outpatient CAP was high among patients without comorbidities but substantially lower among those with comorbidities, with disparities by clinician type, race/ethnicity (comorbidities only), and neighborhood deprivation. These findings reveal systemic inequities in antibiotic stewardship and reinforce the need for equity-focused strategies to support guideline-concordant prescribing. Additional work is warranted to clarify prescribing variation and evaluate relevant modifiers.

**Figure:**